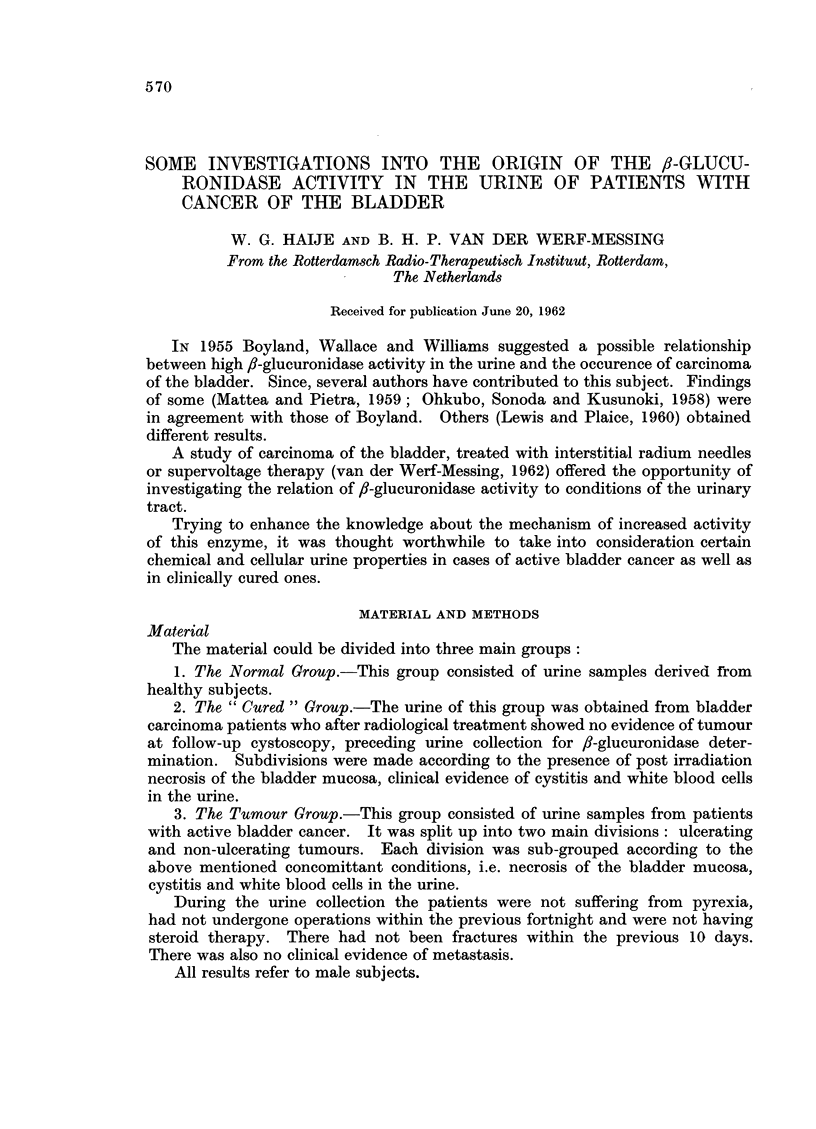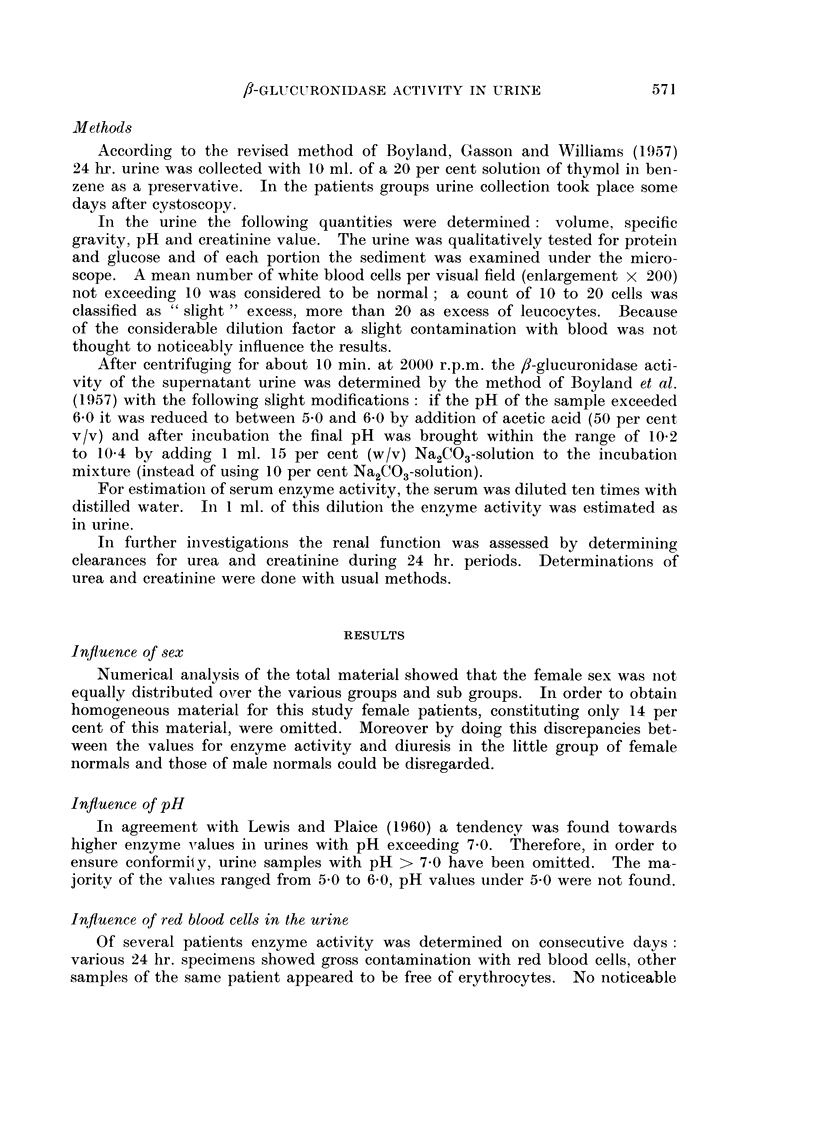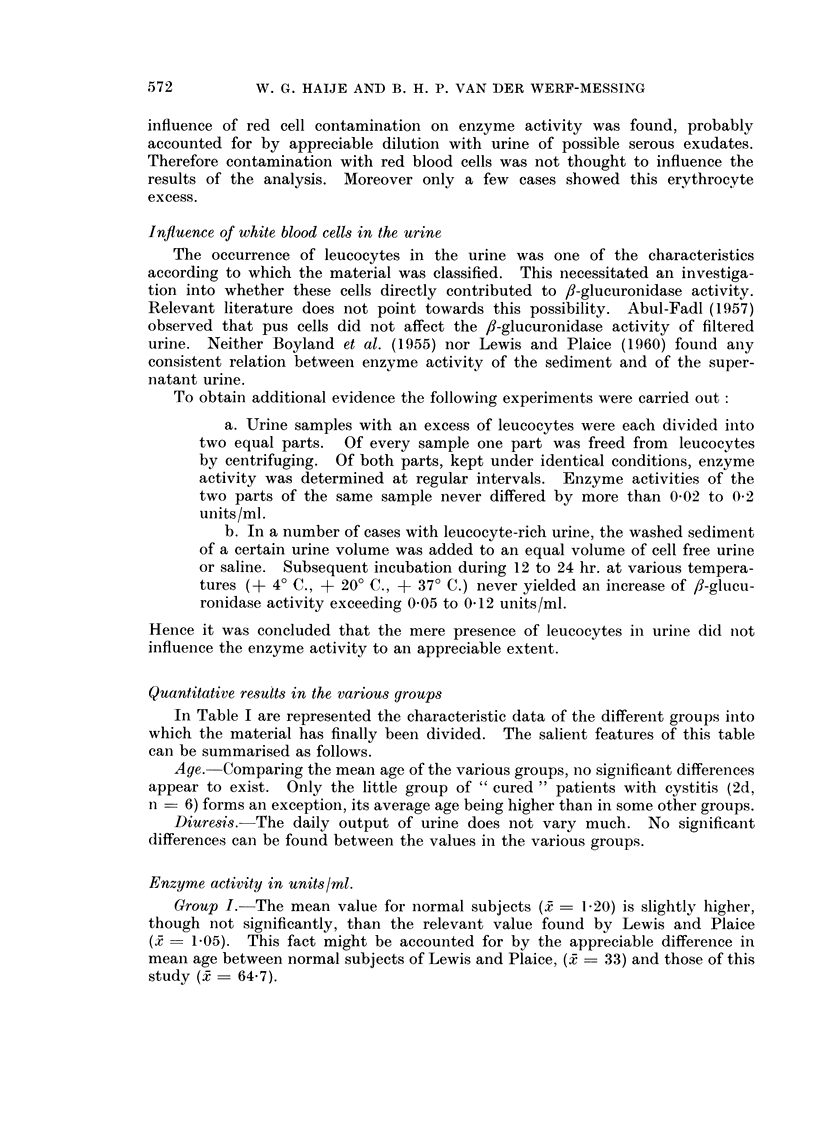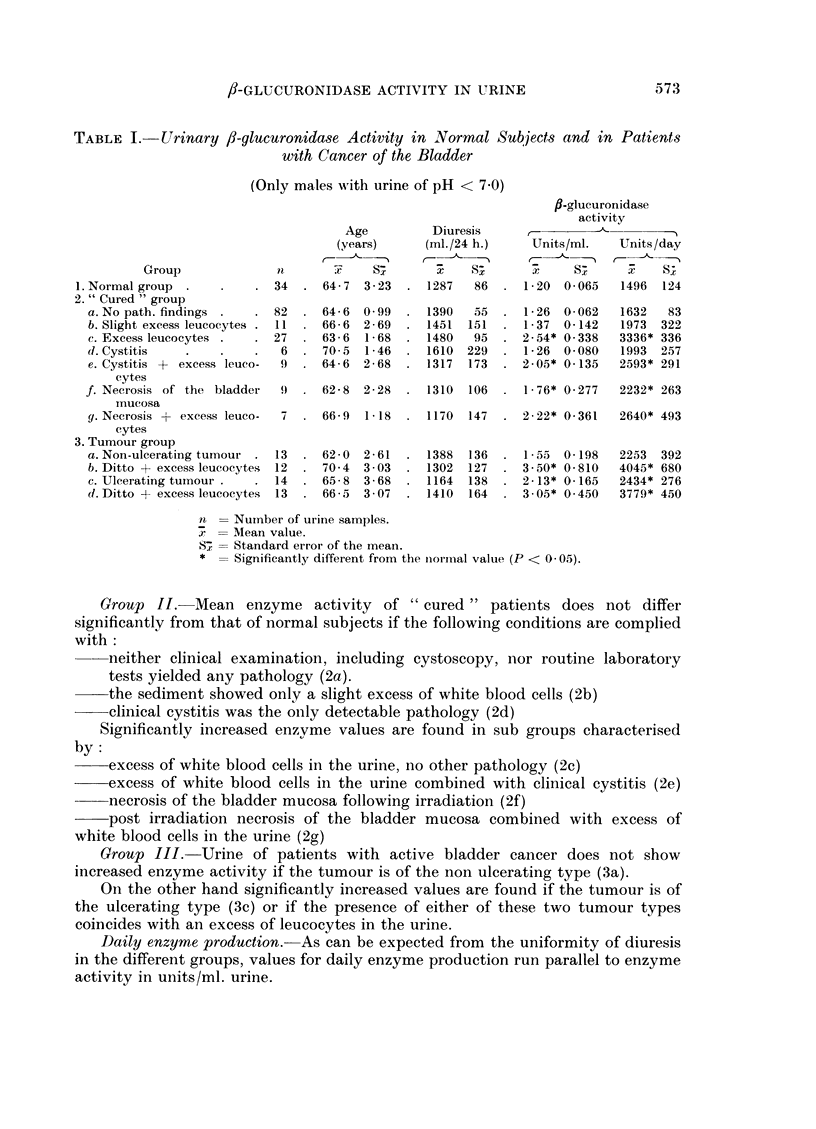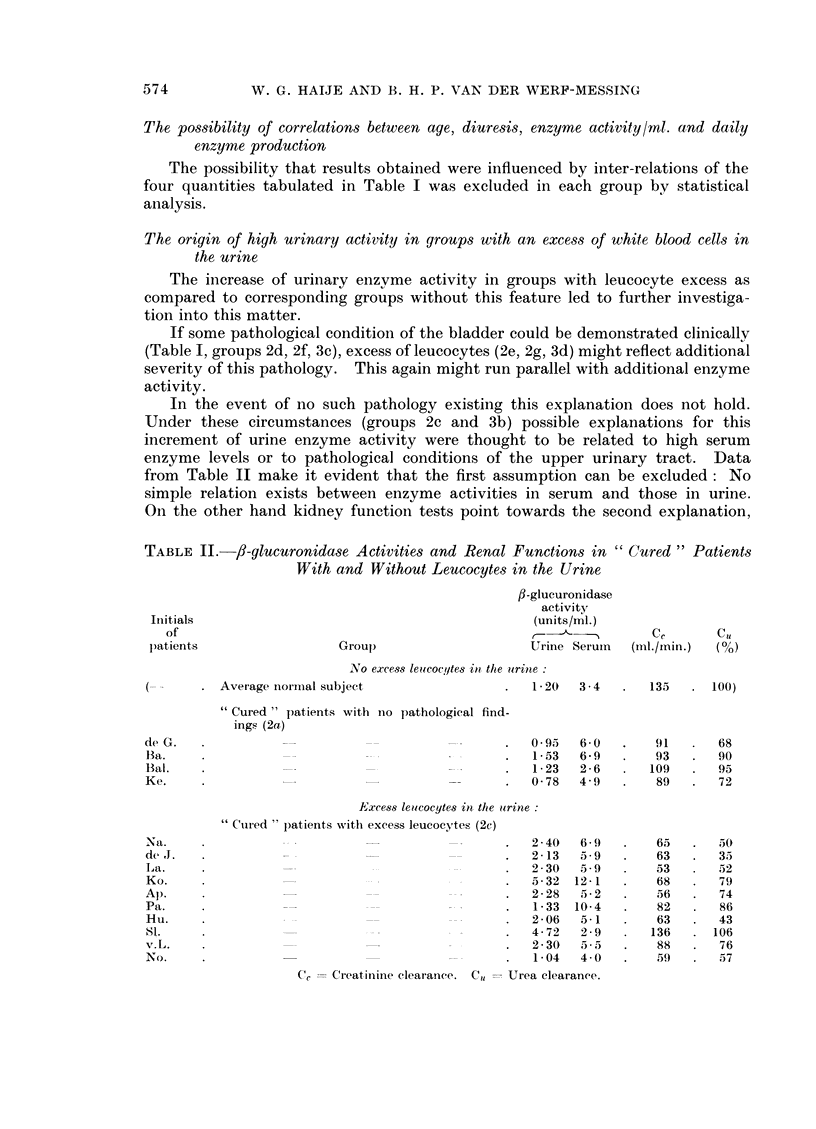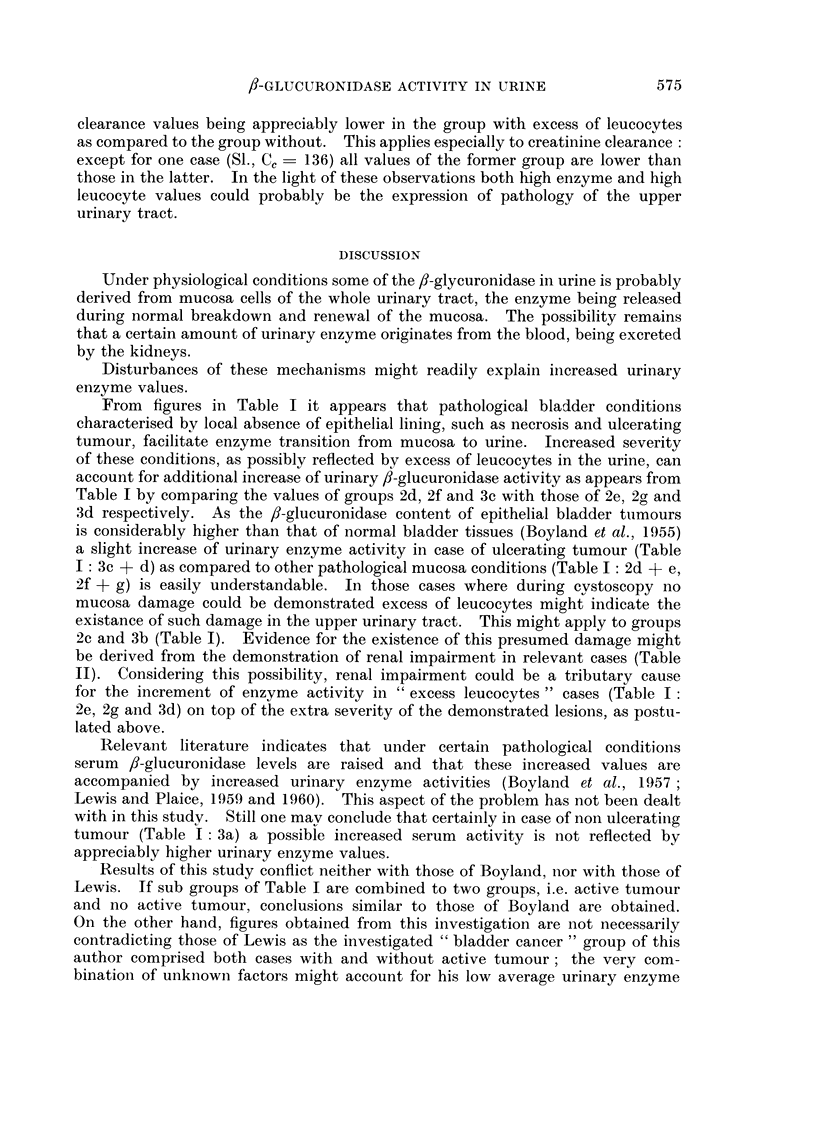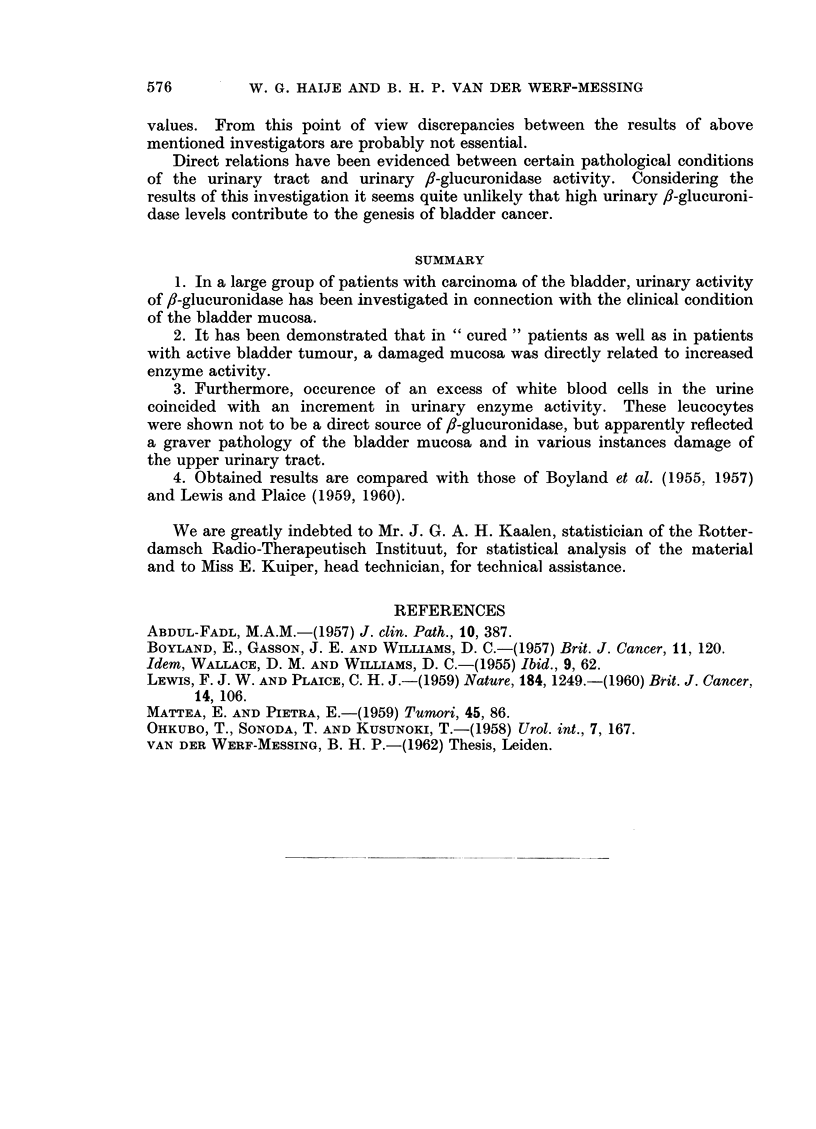# Some Investigations into the Origin of the β-Glucuronidase Activity in the Urine of Patients with Cancer of the Bladder

**DOI:** 10.1038/bjc.1962.66

**Published:** 1962-09

**Authors:** W. G. Haije, B. H. P. van der Werf-Messing


### SOME INVESTIGATIONS INTO THE ORIGIN OF THE $\beta$-GLUCURONIDASE ACTIVITY IN THE URINE OF PATIENTS WITH CANCER OF THE BLADDER

W. G. HAIJE AND B. H. P. VAN DER WERF-MESSING

*From the Rotterdamsch Radio-Therapeutisch Instituut, Rotterdam, The Netherlands*

Received for publication June 20, 1962

IN 1955 Boyland, Wallace and Williams suggested a possible relationship between high $\beta$-glucuronidase activity in the urine and the occurence of carcinoma of the bladder. Since, several authors have contributed to this subject. Findings of some (Mattea and Pietra, 1959; Ohkubo, Sonoda and Kusunoki, 1958) were in agreement with those of Boyland. Others (Lewis and Plaice, 1960) obtained different results.

A study of carcinoma of the bladder, treated with interstitial radium needles or supervoltage therapy (van der Werf-Messing, 1962) offered the opportunity of investigating the relation of $\beta$-glucuronidase activity to conditions of the urinary tract.

Trying to enhance the knowledge about the mechanism of increased activity of this enzyme, it was thought worthwhile to take into consideration certain chemical and cellular urine properties in cases of active bladder cancer as well as in clinically cured ones.

### MATERIAL AND METHODS

### *Material*

The material could be divided into three main groups :

1. *The Normal Group.*—This group consisted of urine samples derived from healthy subjects.

2. *The " Cured " Group.*—The urine of this group was obtained from bladder carcinoma patients who after radiological treatment showed no evidence of tumour at follow-up cystoscopy, preceding urine collection for $\beta$-glucuronidase determination. Subdivisions were made according to the presence of post irradiation necrosis of the bladder mucosa, clinical evidence of cystitis and white blood cells in the urine.

3. *The Tumour Group.*—This group consisted of urine samples from patients with active bladder cancer. It was split up into two main divisions : ulcerating and non-ulcerating tumours. Each division was sub-grouped according to the above mentioned concomittant conditions, i.e. necrosis of the bladder mucosa, cystitis and white blood cells in the urine.

During the urine collection the patients were not suffering from pyrexia, had not undergone operations within the previous fortnight and were not having steroid therapy. There had not been fractures within the previous 10 days. There was also no clinical evidence of metastasis.

All results refer to male subjects.

*Methods*

According to the revised method of Boyland, Gasson and Williams (1957) 24 hr. urine was collected with 10 ml. of a 20 per cent solution of thymol in benzene as a preservative. In the patients groups urine collection took place some days after cystoscopy.

In the urine the following quantities were determined: volume, specific gravity, pH and creatinine value. The urine was qualitatively tested for protein and glucose and of each portion the sediment was examined under the microscope. A mean number of white blood cells per visual field (enlargement $\times$ 200) not exceeding 10 was considered to be normal; a count of 10 to 20 cells was classified as "slight" excess, more than 20 as excess of leucocytes. Because of the considerable dilution factor a slight contamination with blood was not thought to noticeably influence the results.

After centrifuging for about 10 min. at 2000 r.p.m. the β-glucuronidase activity of the supernatant urine was determined by the method of Boyland *et al.* (1957) with the following slight modifications: if the pH of the sample exceeded 6·0 it was reduced to between 5·0 and 6·0 by addition of acetic acid (50 per cent v/v) and after incubation the final pH was brought within the range of 10·2 to 10·4 by adding 1 ml. 15 per cent (w/v) $Na_2CO_3$-solution to the incubation mixture (instead of using 10 per cent $Na_2CO_3$-solution).

For estimation of serum enzyme activity, the serum was diluted ten times with distilled water. In 1 ml. of this dilution the enzyme activity was estimated as in urine.

In further investigations the renal function was assessed by determining clearances for urea and creatinine during 24 hr. periods. Determinations of urea and creatinine were done with usual methods.

### RESULTS

*Influence of sex*

Numerical analysis of the total material showed that the female sex was not equally distributed over the various groups and sub groups. In order to obtain homogeneous material for this study female patients, constituting only 14 per cent of this material, were omitted. Moreover by doing this discrepancies between the values for enzyme activity and diuresis in the little group of female normals and those of male normals could be disregarded.

*Influence of pH*

In agreement with Lewis and Plaice (1960) a tendency was found towards higher enzyme values in urines with pH exceeding 7·0. Therefore, in order to ensure conformity, urine samples with pH $>$ 7·0 have been omitted. The majority of the values ranged from 5·0 to 6·0, pH values under 5·0 were not found.

*Influence of red blood cells in the urine*

Of several patients enzyme activity was determined on consecutive days: various 24 hr. specimens showed gross contamination with red blood cells, other samples of the same patient appeared to be free of erythrocytes. No noticeable influence of red cell contamination on enzyme activity was found, probably accounted for by appreciable dilution with urine of possible serous exudates. Therefore contamination with red blood cells was not thought to influence the results of the analysis. Moreover only a few cases showed this erythrocyte excess.

*Influence of white blood cells in the urine*

The occurrence of leucocytes in the urine was one of the characteristics according to which the material was classified. This necessitated an investigation into whether these cells directly contributed to $\beta$-glucuronidase activity. Relevant literature does not point towards this possibility. Abul-Fadl (1957) observed that pus cells did not affect the $\beta$-glucuronidase activity of filtered urine. Neither Boyland *et al.* (1955) nor Lewis and Plaice (1960) found any consistent relation between enzyme activity of the sediment and of the supernatant urine.

To obtain additional evidence the following experiments were carried out :

a. Urine samples with an excess of leucocytes were each divided into two equal parts. Of every sample one part was freed from leucocytes by centrifuging. Of both parts, kept under identical conditions, enzyme activity was determined at regular intervals. Enzyme activities of the two parts of the same sample never differed by more than 0·02 to 0·2 units/ml.

b. In a number of cases with leucocyte-rich urine, the washed sediment of a certain urine volume was added to an equal volume of cell free urine or saline. Subsequent incubation during 12 to 24 hr. at various temperatures (+ 4° C., + 20° C., + 37° C.) never yielded an increase of $\beta$-glucuronidase activity exceeding 0·05 to 0·12 units/ml.

Hence it was concluded that the mere presence of leucocytes in urine did not influence the enzyme activity to an appreciable extent.

*Quantitative results in the various groups*

In Table I are represented the characteristic data of the different groups into which the material has finally been divided. The salient features of this table can be summarised as follows.

*Age.*—Comparing the mean age of the various groups, no significant differences appear to exist. Only the little group of " cured " patients with cystitis (2d, n = 6) forms an exception, its average age being higher than in some other groups.

*Diuresis.*—The daily output of urine does not vary much. No significant differences can be found between the values in the various groups.

*Enzyme activity in units/ml.*

*Group I.*—The mean value for normal subjects ($\bar{x}$ = 1·20) is slightly higher, though not significantly, than the relevant value found by Lewis and Plaice ($\bar{x}$ = 1·05). This fact might be accounted for by the appreciable difference in mean age between normal subjects of Lewis and Plaice, ($\bar{x}$ = 33) and those of this study ($\bar{x}$ = 64·7).

TABLE I.—*Urinary β-glucuronidase Activity in Normal Subjects and in Patients with Cancer of the Bladder*

(Only males with urine of pH < 7·0)

| Group | $n$ | Age (years) $\bar{x}$ | Age (years) $S_{\bar{x}}$ | Diuresis (ml./24 h.) $\bar{x}$ | Diuresis (ml./24 h.) $S_{\bar{x}}$ | β-glucuronidase activity Units/ml. $\bar{x}$ | Units/ml. $S_{\bar{x}}$ | Units/day $\bar{x}$ | Units/day $S_{\bar{x}}$ |
|---|---|---|---|---|---|---|---|---|---|
| 1. Normal group | 34 | 64·7 | 3·23 | 1287 | 86 | 1·20 | 0·065 | 1496 | 124 |
| 2. " Cured " group | | | | | | | | | |
| *a*. No path. findings | 82 | 64·6 | 0·99 | 1390 | 55 | 1·26 | 0·062 | 1632 | 83 |
| *b*. Slight excess leucocytes | 11 | 66·6 | 2·69 | 1451 | 151 | 1·37 | 0·142 | 1973 | 322 |
| *c*. Excess leucocytes | 27 | 63·6 | 1·68 | 1480 | 95 | 2·54* | 0·338 | 3336* | 336 |
| *d*. Cystitis | 6 | 70·5 | 1·46 | 1610 | 229 | 1·26 | 0·080 | 1993 | 257 |
| *e*. Cystitis + excess leucocytes | 9 | 64·6 | 2·68 | 1317 | 173 | 2·05* | 0·135 | 2593* | 291 |
| *f*. Necrosis of the bladder mucosa | 9 | 62·8 | 2·28 | 1310 | 106 | 1·76* | 0·277 | 2232* | 263 |
| *g*. Necrosis + excess leucocytes | 7 | 66·9 | 1·18 | 1170 | 147 | 2·22* | 0·361 | 2640* | 493 |
| 3. Tumour group | | | | | | | | | |
| *a*. Non-ulcerating tumour | 13 | 62·0 | 2·61 | 1388 | 136 | 1·55 | 0·198 | 2253 | 392 |
| *b*. Ditto + excess leucocytes | 12 | 70·4 | 3·03 | 1302 | 127 | 3·50* | 0·810 | 4045* | 680 |
| *c*. Ulcerating tumour | 14 | 65·8 | 3·68 | 1164 | 138 | 2·13* | 0·165 | 2434* | 276 |
| *d*. Ditto + excess leucocytes | 13 | 66·5 | 3·07 | 1410 | 164 | 3·05* | 0·450 | 3779* | 450 |

$n$ = Number of urine samples.
$\bar{x}$ = Mean value.
$S_{\bar{x}}$ = Standard error of the mean.
* = Significantly different from the normal value ($P < 0·05$).

*Group II*.—Mean enzyme activity of " cured " patients does not differ significantly from that of normal subjects if the following conditions are complied with :

——neither clinical examination, including cystoscopy, nor routine laboratory tests yielded any pathology (2*a*).
——the sediment showed only a slight excess of white blood cells (2b)
——clinical cystitis was the only detectable pathology (2d)

Significantly increased enzyme values are found in sub groups characterised by :

——excess of white blood cells in the urine, no other pathology (2c)
——excess of white blood cells in the urine combined with clinical cystitis (2e)
——necrosis of the bladder mucosa following irradiation (2f)
——post irradiation necrosis of the bladder mucosa combined with excess of white blood cells in the urine (2g)

*Group III*.—Urine of patients with active bladder cancer does not show increased enzyme activity if the tumour is of the non ulcerating type (3a).

On the other hand significantly increased values are found if the tumour is of the ulcerating type (3c) or if the presence of either of these two tumour types coincides with an excess of leucocytes in the urine.

*Daily enzyme production*.—As can be expected from the uniformity of diuresis in the different groups, values for daily enzyme production run parallel to enzyme activity in units/ml. urine.

*The possibility of correlations between age, diuresis, enzyme activity/ml. and daily enzyme production*

The possibility that results obtained were influenced by inter-relations of the four quantities tabulated in Table I was excluded in each group by statistical analysis.

*The origin of high urinary activity in groups with an excess of white blood cells in the urine*

The increase of urinary enzyme activity in groups with leucocyte excess as compared to corresponding groups without this feature led to further investigation into this matter.

If some pathological condition of the bladder could be demonstrated clinically (Table I, groups 2d, 2f, 3c), excess of leucocytes (2e, 2g, 3d) might reflect additional severity of this pathology. This again might run parallel with additional enzyme activity.

In the event of no such pathology existing this explanation does not hold. Under these circumstances (groups 2c and 3b) possible explanations for this increment of urine enzyme activity were thought to be related to high serum enzyme levels or to pathological conditions of the upper urinary tract. Data from Table II make it evident that the first assumption can be excluded : No simple relation exists between enzyme activities in serum and those in urine. On the other hand kidney function tests point towards the second explanation,

TABLE II.—*β-glucuronidase Activities and Renal Functions in " Cured " Patients With and Without Leucocytes in the Urine*

| Initials of patients | Group | | | β-glucuronidase activity (units/ml.) Urine | Serum | $C_c$ (ml./min.) | $C_u$ (%) |
|---|---|---|---|---|---|---|---|
| | *No excess leucocytes in the urine :* | | | | | | |
| (— | Average normal subject | | | 1·20 | 3·4 | 135 | 100) |
| | " Cured " patients with no pathological findings (2*a*) | | | | | | |
| de G. | — | — | — | 0·95 | 6·0 | 91 | 68 |
| Ba. | — | — | — | 1·53 | 6·9 | 93 | 90 |
| Bal. | — | — | — | 1·23 | 2·6 | 109 | 95 |
| Ke. | — | — | — | 0·78 | 4·9 | 89 | 72 |
| | *Excess leucocytes in the urine :* | | | | | | |
| | " Cured " patients with excess leucocytes (2*c*) | | | | | | |
| Na. | — | — | — | 2·40 | 6·9 | 65 | 50 |
| de J. | — | — | — | 2·13 | 5·9 | 63 | 35 |
| La. | — | — | — | 2·30 | 5·9 | 53 | 52 |
| Ko. | — | — | — | 5·32 | 12·1 | 68 | 79 |
| Ap. | — | — | — | 2·28 | 5·2 | 56 | 74 |
| Pa. | — | — | — | 1·33 | 10·4 | 82 | 86 |
| Hu. | — | — | — | 2·06 | 5·1 | 63 | 43 |
| Sl. | — | — | — | 4·72 | 2·9 | 136 | 106 |
| v.L. | — | — | — | 2·30 | 5·5 | 88 | 76 |
| No. | — | — | — | 1·04 | 4·0 | 59 | 57 |

$C_c$ = Creatinine clearance. $C_u$ = Urea clearance.

clearance values being appreciably lower in the group with excess of leucocytes as compared to the group without. This applies especially to creatinine clearance : except for one case (Sl., $C_c = 136$) all values of the former group are lower than those in the latter. In the light of these observations both high enzyme and high leucocyte values could probably be the expression of pathology of the upper urinary tract.

### DISCUSSION

Under physiological conditions some of the β-glycuronidase in urine is probably derived from mucosa cells of the whole urinary tract, the enzyme being released during normal breakdown and renewal of the mucosa. The possibility remains that a certain amount of urinary enzyme originates from the blood, being excreted by the kidneys.

Disturbances of these mechanisms might readily explain increased urinary enzyme values.

From figures in Table I it appears that pathological bladder conditions characterised by local absence of epithelial lining, such as necrosis and ulcerating tumour, facilitate enzyme transition from mucosa to urine. Increased severity of these conditions, as possibly reflected by excess of leucocytes in the urine, can account for additional increase of urinary β-glucuronidase activity as appears from Table I by comparing the values of groups 2d, 2f and 3c with those of 2e, 2g and 3d respectively. As the β-glucuronidase content of epithelial bladder tumours is considerably higher than that of normal bladder tissues (Boyland *et al.*, 1955) a slight increase of urinary enzyme activity in case of ulcerating tumour (Table I : 3c + d) as compared to other pathological mucosa conditions (Table I : 2d + e, 2f + g) is easily understandable. In those cases where during cystoscopy no mucosa damage could be demonstrated excess of leucocytes might indicate the existance of such damage in the upper urinary tract. This might apply to groups 2c and 3b (Table I). Evidence for the existence of this presumed damage might be derived from the demonstration of renal impairment in relevant cases (Table II). Considering this possibility, renal impairment could be a tributary cause for the increment of enzyme activity in " excess leucocytes " cases (Table I : 2e, 2g and 3d) on top of the extra severity of the demonstrated lesions, as postulated above.

Relevant literature indicates that under certain pathological conditions serum β-glucuronidase levels are raised and that these increased values are accompanied by increased urinary enzyme activities (Boyland *et al.*, 1957 ; Lewis and Plaice, 1959 and 1960). This aspect of the problem has not been dealt with in this study. Still one may conclude that certainly in case of non ulcerating tumour (Table I : 3a) a possible increased serum activity is not reflected by appreciably higher urinary enzyme values.

Results of this study conflict neither with those of Boyland, nor with those of Lewis. If sub groups of Table I are combined to two groups, i.e. active tumour and no active tumour, conclusions similar to those of Boyland are obtained. On the other hand, figures obtained from this investigation are not necessarily contradicting those of Lewis as the investigated " bladder cancer " group of this author comprised both cases with and without active tumour ; the very combination of unknown factors might account for his low average urinary enzyme values. From this point of view discrepancies between the results of above mentioned investigators are probably not essential.

Direct relations have been evidenced between certain pathological conditions of the urinary tract and urinary $\beta$-glucuronidase activity. Considering the results of this investigation it seems quite unlikely that high urinary $\beta$-glucuronidase levels contribute to the genesis of bladder cancer.

### SUMMARY

1. In a large group of patients with carcinoma of the bladder, urinary activity of $\beta$-glucuronidase has been investigated in connection with the clinical condition of the bladder mucosa.

2. It has been demonstrated that in " cured " patients as well as in patients with active bladder tumour, a damaged mucosa was directly related to increased enzyme activity.

3. Furthermore, occurence of an excess of white blood cells in the urine coincided with an increment in urinary enzyme activity. These leucocytes were shown not to be a direct source of $\beta$-glucuronidase, but apparently reflected a graver pathology of the bladder mucosa and in various instances damage of the upper urinary tract.

4. Obtained results are compared with those of Boyland *et al.* (1955, 1957) and Lewis and Plaice (1959, 1960).

We are greatly indebted to Mr. J. G. A. H. Kaalen, statistician of the Rotterdamsch Radio-Therapeutisch Instituut, for statistical analysis of the material and to Miss E. Kuiper, head technician, for technical assistance.

### REFERENCES

Abdul-Fadl, M.A.M.—(1957) *J. clin. Path.*, **10**, 387.
Boyland, E., Gasson, J. E. and Williams, D. C.—(1957) *Brit. J. Cancer*, **11**, 120.
*Idem*, Wallace, D. M. and Williams, D. C.—(1955) *Ibid.*, **9**, 62.
Lewis, F. J. W. and Plaice, C. H. J.—(1959) *Nature*, **184**, 1249.—(1960) *Brit. J. Cancer*, **14**, 106.
Mattea, E. and Pietra, E.—(1959) *Tumori*, **45**, 86.
Ohkubo, T., Sonoda, T. and Kusunoki, T.—(1958) *Urol. int.*, **7**, 167.
van der Werf-Messing, B. H. P.—(1962) Thesis, Leiden.